# Discriminating TB lung nodules from early lung cancers using deep learning

**DOI:** 10.1186/s12911-022-01904-8

**Published:** 2022-06-21

**Authors:** Heng Tan, Jason H. T. Bates, C. Matthew Kinsey

**Affiliations:** 1 Department of Medicine, Larner College of Medicine, University of Vermont, Burlington, VT USA; 2 Interventional Pulmonary, University of Vermont Medical Center, Burlington, VT USA

**Keywords:** Lung cancer, Latent TB, Deep learning

## Abstract

**Background:**

In developing countries where both high rates of smoking and endemic tuberculosis (TB) are often present, identification of early lung cancer can be significantly confounded by the presence of nodules such as those due to latent TB (LTB). It is very challenging to distinguish lung cancer and LTB without invasive procedures, which have their own risks of morbidity and even mortality.

**Methods:**

Our method uses a customized VGG16-based 15-layer 2-dimensional deep convolutional neural network (DNN) architecture with transfer learning. The DNN was trained and tested on sets of CT images set extracted from the National Lung Screening Trial and the National Institute of Allergy and Infectious Disease TB Portals. Performance of the DNN was evaluated under locked and step-wise unlocked pretrained weight conditions.

**Results:**

The DNN with unlocked pretrained weights achieved an accuracy of 90.4% with an F score of 90.1%.

**Conclusions:**

Our findings support the potential for a DNN to serve as a noninvasive screening tool capable of reliably detecting and distinguishing between lung cancer and LTB.

## Background

One of the most common communicable causes of morbidity and mortality, pulmonary tuberculosis (TB), has been classified by the World Health Organization (WHO) as a global public health emergency since 1993 [1]. According to the 2020 WHO report there were an estimated 10 million symptomatic individuals with TB worldwide of whom 1.4 million died [2], with nearly 70% of those suffering from the infection living in South-East Asia and Africa. Most of the morbidity and mortality related to TB is due to reactivation of the disease, which accounts for approximately 80% of all active TB cases [3] and is responsible for most disease spread. Nevertheless, although TB remains the leading cause of death among infectious diseases, it falls well below cancer in terms of total mortality [4]. Lung cancer is the most common cause of cancer-related death both in the United States and worldwide [5]. Most lung cancers (85–90%) are classified as non-small cell lung cancer (NSCLC), which is highly correlated with smoking and has a survival rate that is dramatically affected by the stage at detection. In contrast to developed nations where the incidence of smoking is falling, cigarette smoking is on the rise in developing nations many of whom have a high rate of endemic tuberculosis. In the 16 low and middle-income countries participating in the Global Adult Tobacco Survey, representing more than half of the world's smokers, the active smoking rates were as high as 67% in men and 29% in women [6]. Ten of these countries also appear on the WHO high-burden tuberculosis list [2].

In countries with both high rates of smoking and endemic tuberculosis, identification of early lung cancer can be significantly confounded by the presence of lung nodules due to latent TB (LTB). Unfortunately, these two entities cannot be readily distinguished even by trained radiologists. This diagnostic equipoise leads to significant delays in cancer diagnosis, a disease for which timely intervention is paramount, with concommitant increases in lung cancer mortality [7]. Treatment options for lung cancer are also very different than those for TB. Accordingly, there is a critical need for improved methods of distinguishing between TB and lung cancer in the classification of suspicious lung nodules seen on CT.

This is a problem that would seem to be ideally suited to machine learning, and indeed a number of previous studies have taken this approach to the segmentation of lung nodule images [8–11], the detection of TB nodules [12–14], and classification of lung nodules as either malignant or benign [15–18]. Little work has been done, however, on the use of machine learning to discriminate between TB and lung cancer. Feng et al. [19] was able to train a deep neural network (DNN) to classify TB granulomas versus lung adenocarcinomas with an accuracy of up to 81%, but adenocarcinomas represent less than half of all lung cancers. The goal of the present study, therefore, was to develop a DNN capable of differentiating TB from lung cancer in general. We trained and tested the DNN on two large data sets, one taken from the National Lung Screening Trial and the other from the National Institutes of Allergy and Infectious Disease Tuberculosis Portal.

## Materials and methods

### Lung nodule datasets

**Fig. 1:**
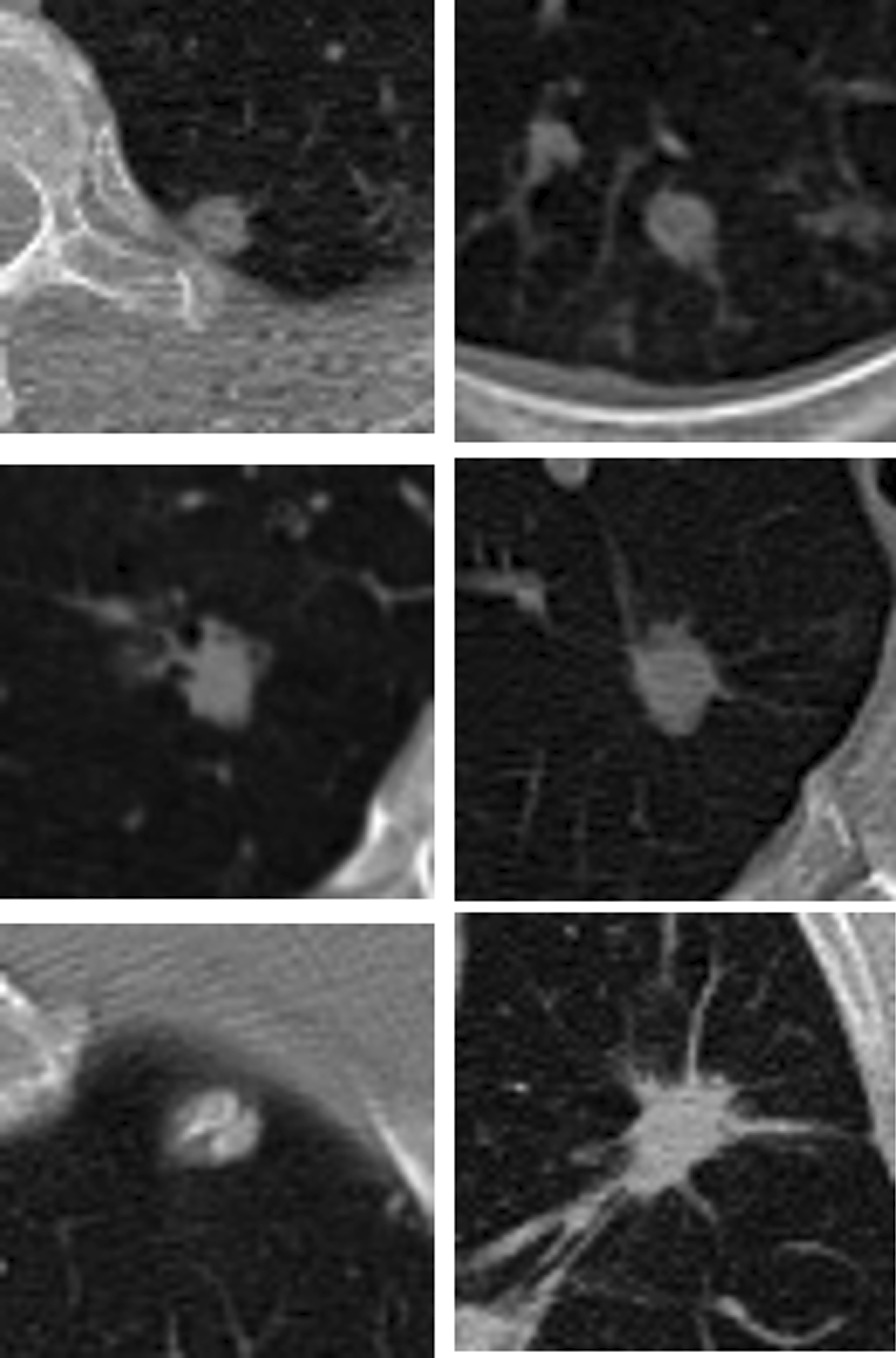
Examples of nodules undergoing classification. All nodules in the left sided panels are malignant, while the nodules in the right sided panels are tuberculous. The tuberculous nodule in the bottom right is morphologically “spiculated”, a characteristic typically associated with malignancy

De-identified data from the National Lung Screening Trial (NLST) and the National Institute of Allergy and Infectious Disease (NIAID) TB Portal were evaluated under separate data-use agreements. All methods involved in the collection of these data were performed in accordance with the relevant guidelines and regulations. These date sets were individually approved as not requiring additional approval by the Research Protections Office of the University of Vermont. We reviewed 297 CT scans from the NIAID dataset and selected 172 images with the same lung convolution kernel and slice width of 2.5 mm in order to ensure consistent image quality. We used 3D Slicer software [20] to identify 436 separate 2D axial images of nodules having diameters between 6 and 30 mm. The lower end of this range, 6 mm, represents the smallest nodules that have clinical importance in terms of lung malignancy and that thus require further investigation. The nodule images were cropped from each CT slice using a Python script and saved into 64 × 64 pixel gray scale images in JPG format. The images in the NLST dataset were processed similarly, yielding 517 malignant nodules cropped from 517 CT scans. Figure 1 shows two examples of cropped images, one malignant and one benign. Of the 953 nodules included in the study, 65% were used for training the DNN, 10% for validation, and 25% for testing.

### DNN architecture

Due to the limited size of the image dataset at our disposal, we implemented a transfer learning methodology [21] that re-utilized weights determined by prior training on a very large but unrelated dataset. A customized VGG16 [22] network architecture was adapted as the testing platform. The weights in the convolutional layers of this architecture had been pre-trained using a dataset of over 14 million images belonging to 1000 disparate classes, which allowed it to achieve 92.7% accuracy placing it in the top 5 ImageNet performers. The input layer of our DNN was an array of size 3 × 64 × 64, designed to it could receive 3-channel RGB images. Since our nodule images had a single gray scale channel, we duplicated the gray scale array 3 times to match the input format of the architecture. The input layer was followed by 13 convolutional layers and 4 pooling layers. For each convolutional layer, the kernel size was 3 × 3 with a stride of 1 pixel. The output of each convolutional layer feds into an activation layer equipped with a rectified linear unit (RELU) activation function such that RELU(x) = Max(0,x), meaning that it simply replaced any negative values with zero. The pooling layers down-sample the output of the convolution layers over a 2 × 2 pixel window, with stride of 2. The customized VGG16 model consisting of two fully connected layers that served as the multi-layer perceptron classifier. The two output nodes of the VGG16 yielded the final decision probabilities of TB versus malignancy.

The inferences made by the DNN were visualized by Gradient-weighted Class Activation Mapping (Grad-CAM) [23] from two perspectives: (1) visualization of the existing pixel-space features learned by the convolutional layers, and (2) visualization of the decision-making process as shown by the class-specific gradient information flowing into the final convolutional layer of the DNN to produce two types of coarse localization maps, including heat and saliency map, of the important regions in the image. The detailed Grad-CAM frame diagram can be found in Selvaraju et al. [23].

Our DNN was developed in Python code with the Keras package and Tensor Flow. Keras can leverage graphical processing units to accelerate deep learning algorithms. The DNN was trained on a NVIDIA nvdia 2080 ti Graphic Card.

## Results

We first locked the pretrained convolutional layers in the DNN (i.e., the convolutional weights were not allowed to change during training) and found that the following hyperparameter choices yielded the best training performance: (a) 512 hidden neurons in each fully connected layer versus 256 or 64 hidden neurons; (b) an ADAM optimizer versus SGD; (c) a learning rate of 0.001 versus 0.01 or 0.001; (d) a mini-batch gradient descent parameter adjustment scheme with a batch size of 16 versus 32 or higher; (e) no dropout in the fully-connected layers; (f) use of average pooling of the output from the convolutional layers prior to input to the fully connected layers versus max pooling; and (g) no image data preprocessing and augmentation for either the training or validation dataset. These hyperparameter choices were evaluated based on accuracy and loss performance in training, number of epoch rounds for converge in training, and performance results in testing. Figure 5 shows an example of the evaluation result of ADAM optimizer versus SGD optimizer.

**Fig. 2:**
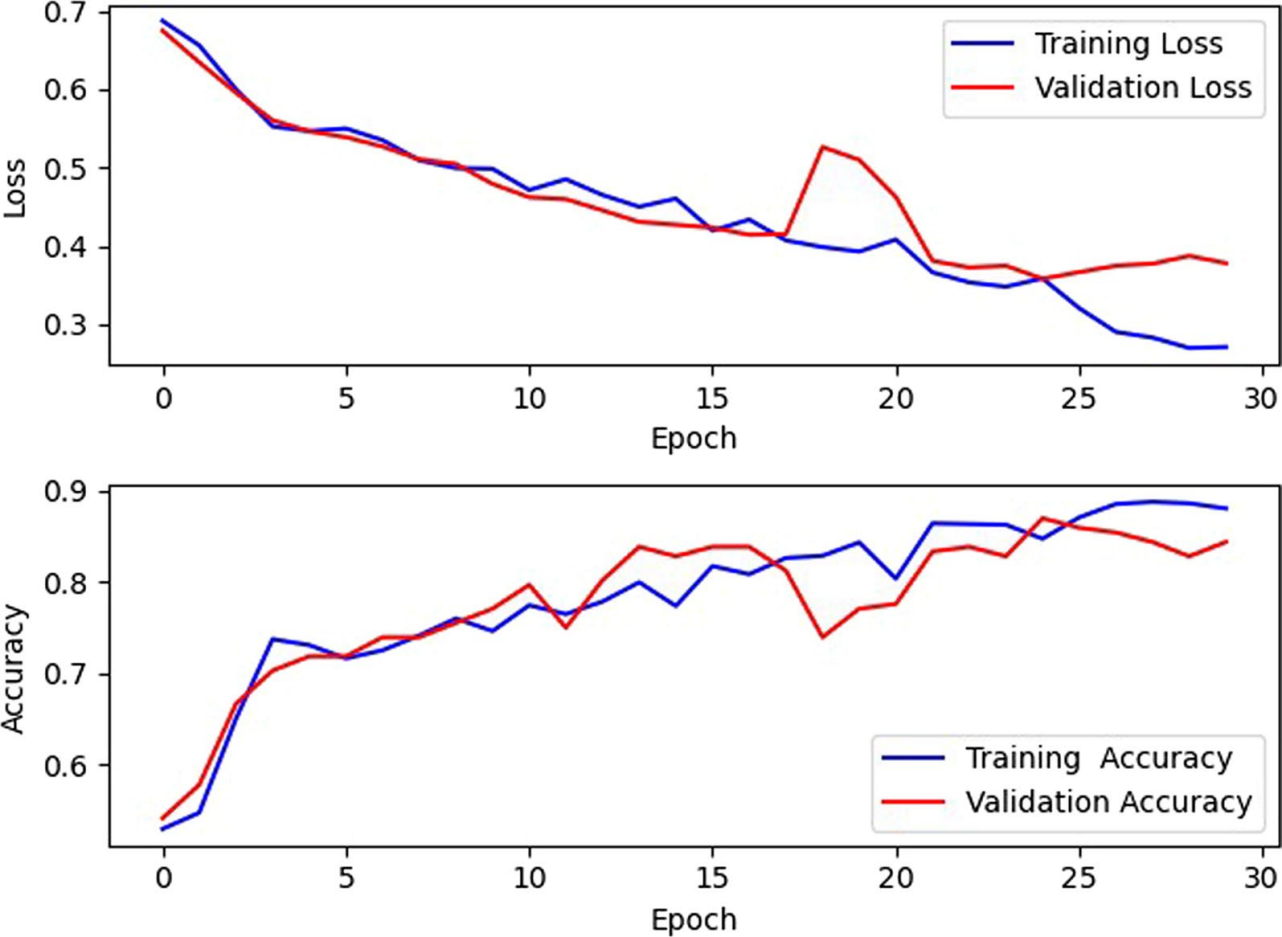
Training and validation performance

**Fig. 3:**
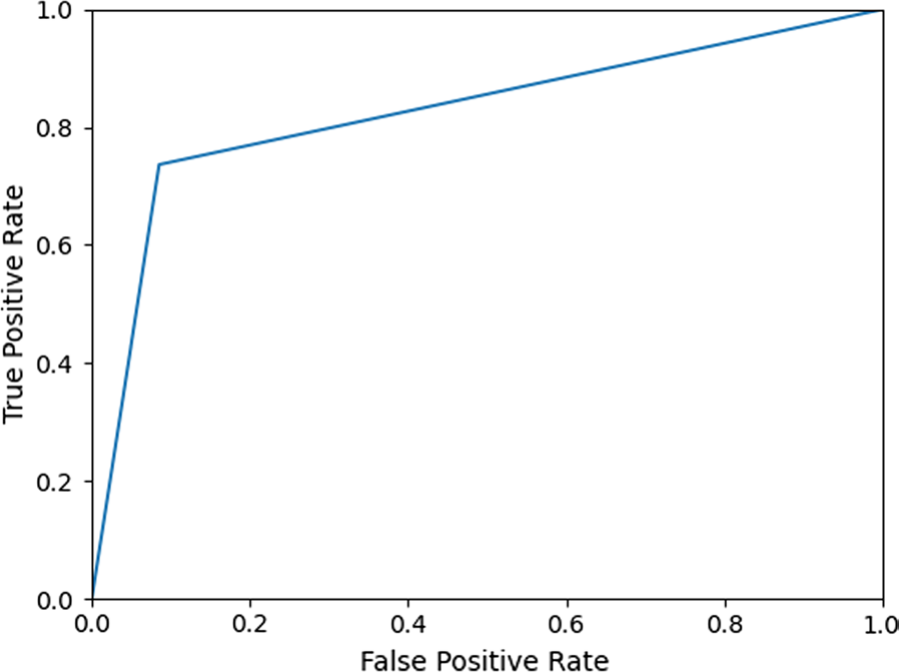
ROC Curve

Figure 2 shows the training loss and accuracy of our DNN during both training (on 65% of the data) and cross-validation (on 10% of the data) after the hyperparameters were optimized and demonstrates that learning was characterized by steady improvement over multiple iterations. Training loss decreased toward 0.2 after 30 epochs, while accuracy increased to nearly 0.9. Near the end of the training process, however, the validation loss and accuracy started to fluctuate, which is a sign of overfitting. Figure 3 shows the receiver operating characteristic (ROC) curve. The area under the curve (AUC) is 0.871.

Our transfer learning methodology was then further evaluated by step-wise unlocking of the pre-trained weights in the convolutional layers. The unlocking process began with the last and most abstract convolutional layer, followed the second-to-last layer, and so on. The DNN was retrained on 70% of the data after each unlocking step, and the following metrics were evaluated using the remaining 30% test dataset: (a) accuracy; (b) precision; (c) sensitivity; (d) specificity; (e) F-score; and (f) AUC. The results are shown in Table 1. Accuracy and AUC steadily improved as each additional layer was unlocked, the greatest improvements being obtained with the first 10 of the 16 layers. The changes in precision, sensitivity and specificity were not consistent nor monotonic with the numb er of unlocked layers, while balance between precision and sensitivity encapsulated by the F-score increased initially but then decreased in going from 10 to 15 unlocked layers. These findings indicate that robustness and generalization ability of transfer learning improves when the weights in the more abstract downstream convolutional layers are allowed to respond to the data, whereas re-tuning pretrained weights in the first 5 layers, which extract simple features, does not improve performance.

**Table 1:** Transfer learning performance metrics with unlocked weight on testing dataset

# of Unlocked layers	Accuracy	Precision	Sensitivity	Specificity	F1	AUC
0	0.875	0.892	0.827	0.915	0.858	0.871
5	0.883	0.866	0.882	0.884	0.874	0.883
10	0.904	0.854	0.955	0.860	0.901	0.908
15	0.908	0.931	0.864	0.946	0.896	0.905

**Fig. 4:**
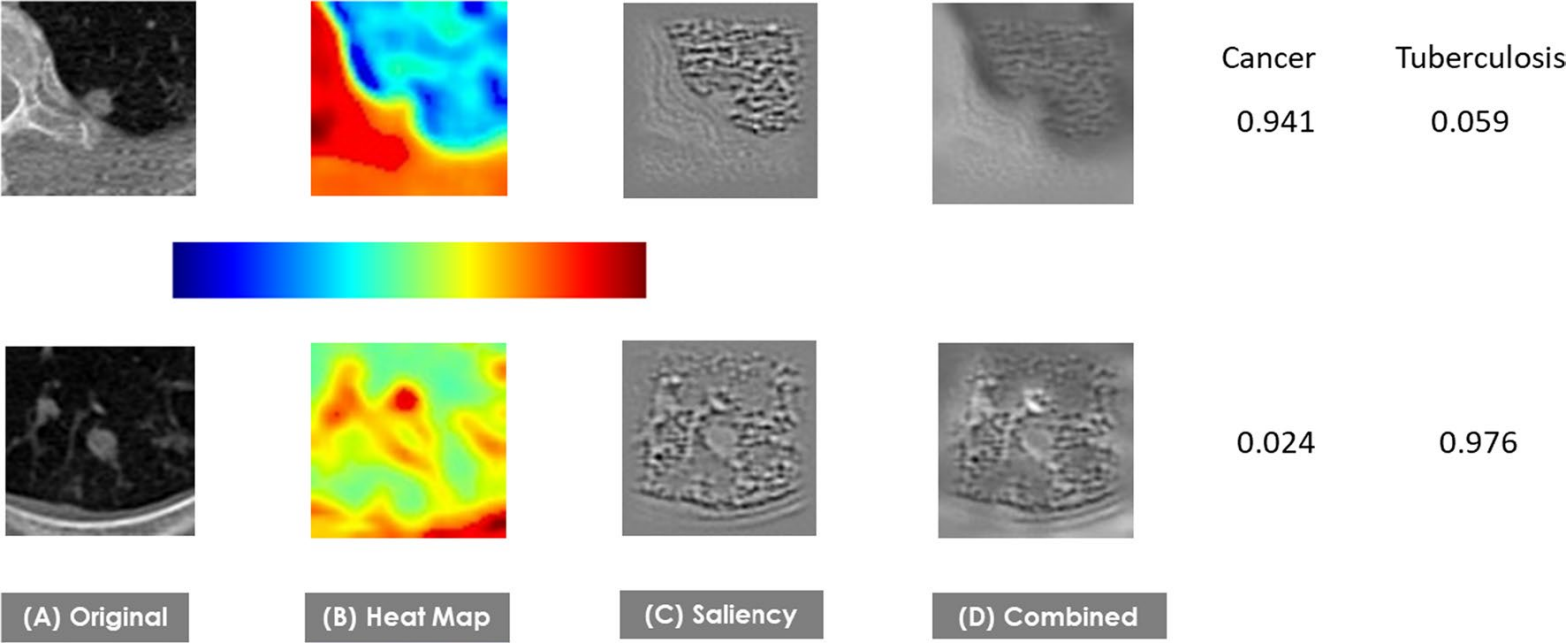
GRAD CAM Visualization. Subplot **A** is the original image; **B** is the heatmap, **C** is the saliency and **D** is the combination of heatmap and Saliency result. Coloring toward the red indicates a higher importance for the final classification. The predicted diagnosis probabilities for cancer versus TB are shown on the right

Figure 4 indicates the image features that were important to decision making for a cancerous nodule and a LTB nodule, as shown by GRAD-CAM. The attention heat maps shown in Fig. 4B highlight areas of importance. In Fig. 4C, the decision processes are also traced back from the last to the first convolutional layer to highlight all edge-related features, known as Saliency. Figure 4D further demonstrates the association between pixel-space features in the images and the diagnostic decisions made by the DNN by combining the heat maps and the saliencies to produce images that highlight the contributing abstract features. The DNN correctly identified the top image as cancer and the bottom image as TB.

## Discussion

DNN’s typically have very large numbers of adjustable weights that must be evaluated through training before achieving reliable classification. Machine learning algorithms thus normally require training with a correspondingly large number of exemplars, sometimes in the millions. Furthermore, training requirements increase exponentially with as the size of the neural network architecture increases in breadth and depth. In the medical field, this can present a significant challenge because collecting large amounts of annotated training data is often expensive, time-consuming, and possibly even unrealistic. We attempted to meet this challenge in the present study by utilizing a customized VGG16 model with a transfer learning methodology in order to differentiate malignant from tuberculous lung nodules. (More complicated deep learning models, such as VGG19 or google net, were not selected because they require even larger training data sets than VGG 16). The major advantage of this approach is that it allows relatively rapid convergence to an adequately trained set of weights with a relatively small dataset. Training our DNN architecture starting with randomly assigned weights would require hundreds of thousands to millions of nodule images, a bar that cannot currently be met.

**Fig. 5:**
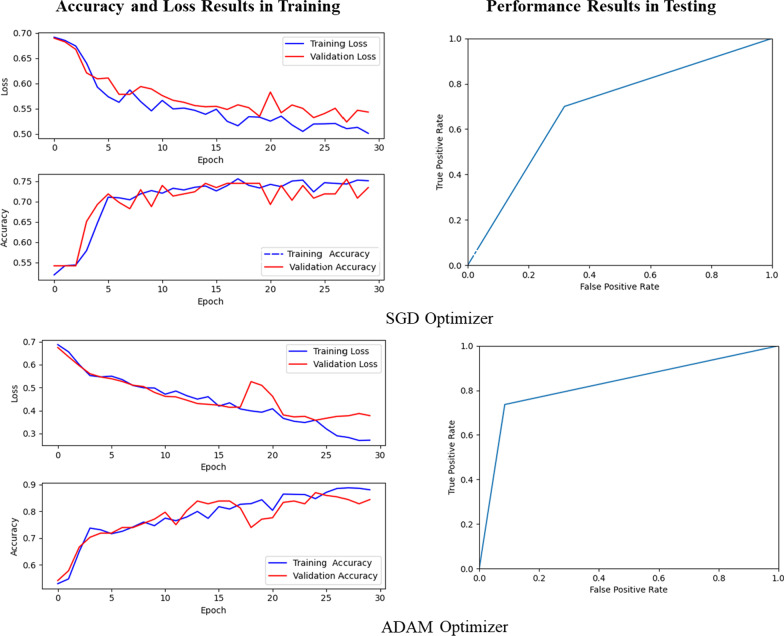
SGD versus ADAM optimizer performance evaluation

Despite our limited dataset, however, using a pre-trained DNN with locked convolutional weights resulted in impressive levels of accuracy, precision, sensitivity, specificity, and F-score (Figs. 2, 3, Table 1). Using a smaller dataset of only 100 images, Feng et al. [19], differentiated tuberculous granulomas from lung adenocarcinomas using approximately 100 example images and achieved an AUC on external validation of 0.809. Our data set was significantly larger, being drawn from multiple sources and inclusive of all malignant subtypes. Despite the greater data variance our DNN was able to achieve an AUC of up to 0.908. This demonstrates the strong potential for machine learning to function as a noninvasive diagnostic tool for differentiation between tuberculous and malignant lung nodules (Fig. 5).

Progressively unlocking the weights of the convolutional layers, beginning with the most distal, led to consistent improvements in AUC and accuracy, although this was most pronounced early on in the process. The downstream layers serve to extract increasingly abstract and complex image features that are likely more specific to the images being classified, so allowing these layers to be trained on the target images presumably leverages this specificity. The earlier convolution layers, in contrast, focus on more primitive features such as lines and simple angles that are likely common to images in general, so little is lost in pre-training these layers on arbitrary datasets. In our case, the optimal balance between allowing specialized training and reducing training time appears to have been achieved by locking the first 6 convolution layers (Table 1).

During the hyperparameter optimization process, we noticed that random combinations of image augmentation techniques such as rotations, horizontal and vertical flipping, or inversions negatively impacted the performance of the model. This is the opposite behavior from what one would normally expect from DNN training [24]. However the precise shape of a lung nodule as well as its orientation with respect to surrounding tissue structures are features that are often key for distinguishing between classifications. It is therefore possible that conventional augmentation operations distorted the information inherent in these features to an extent that confounded the classifier. In any case, these findings suggest that image augmentation techniques should be used sparingly, if at all, for medical image preprocessing when datasets are small.

This study has several notable limitations. First, the study was retrospective and thus prone to selection bias, particularly since the data from the NIAID were acquired as a convenience set. Second, the NIAID dataset contained both active and latent TB cases so, even though LTB would have been in the majority, our results may have been affected by the presence of active TB nodules. Third, we only utilized axial CT slices of nodules, which neglects any information specific to slices at other orientations and possibly also limits generalizability to these orientations. Fourth, despite using the largest dataset we could find, there was still only a limited numbers of exemplars compared to the huge number of adjustable parameters in the DNN. Fifth, we did not include benign nodules in our dataset so we do not know how such nodules would confound the classification of cancer versus TB, nor how successfully deep learning would be able to automatically segment such nodules in CT images. This last point goes beyond the scope of the present study but would be a good area for future investigation. Lastly, although the method we have developed performed extremely well, it nevertheless misclassified some nodules. Visual inspection did not reveal any obvious reasons why this happened, so it presumably reflects the feature overlap that can occur between TB and malignant nodules, which speaks to the inherent difficulty of this classification problem.

## Conclusions

The problem of differentiating between tuberculous and malignant lung nodules in CT images is amenable to the discriminating ability of a deep convolutional neural network as evidenced by the accuracy of 90.8% achieved in the present study. The challenges posed by the inevitably limited size of the training dataset can be mitigated by transfer learning applied to the early convolutional layers; training the later layers on the target dataset imbues the network with the specificity required for optimum performance. Machine learning can thus be a noninvasive and effective tool for clinicians.

## Data Availability

The lung cancer database analyzed in this study is from NIH NLST Portal, https://cdas.cancer.gov/nlst/. The TB database analyzed in this study is from NIH NIAID TB Portal, https://tbportals.niaid.nih.gov/. Both datasets are publicly available after registration and approval from the NIH.

## Abbreviations

LTB: Latent tuberculosis
DNN: Deep neural network
CNN: Convolutional neural network
CT: Computed tomography
ReLU: Rectified linear unit
WHO: World Health Organization
NSCLC: Non-small cell lung cancer
NLST: National Lung Screening Trial
NIAID: National Institute of Allergy and Infectious Disease
Grad-CAM: Gradient-weighted class activation mapping
ROC: Receiver operating characteristic
AUC: Area under the curve
